# Optimizing extrusion processes and understanding conformational changes in itraconazole amorphous solid dispersions using in-line UV–Vis spectroscopy and QbD principles

**DOI:** 10.1016/j.ijpx.2024.100308

**Published:** 2024-11-26

**Authors:** Hetvi Triboandas, Mariana Bezerra, Juan Almeida, Matheus de Castro, Bianca Aloise Maneira Corrêa Santos, Walkiria Schlindwein

**Affiliations:** a Leicester School of Pharmacy, De Montfort University, Leicester LE1 9BH, UK; b GlaxoSmithKline, David Jack Centre, Harris Lane, Ware, Hertfordshire SG12 0GX, UK; c Applied Materials, Daresbury WA4 4AB, UK

**Keywords:** Amorphous Solid Dispersion (ASD), Hot-Melt Extrusion (HME), Itraconazole (ITZ), Quality by Design (QbD), In-line UV–Vis spectroscopy, Density Functional Theory (DFT), Design of Experiments (DoE)

## Abstract

This paper presents a comprehensive investigation of the manufacturing of itraconazole (ITZ) amorphous solid dispersions (ASDs) with Kolllidon® VA64 (KVA64) using hot-melt extrusion (HME) and in-line process monitoring, employing a Quality by Design (QbD) approach. A sequential Design of Experiments (DoE) strategy was utilized to optimize the manufacturing process, with in-line UV–Vis spectroscopy providing real-time monitoring. The first DoE used a fractional factorial screening design to evaluate critical process parameters (CPPs), revealing that ITZ concentration had the most significant impact on the product quality attributes. The second DoE, employing a central composite design, explored the interactions between feed rate and screw speed, using torque and absorbance at 370 nm as responses to develop a design space. Validation studies confirmed process robustness across multiple days, with stable in-line UV–Vis spectra and consistent product quality using 30 % ITZ, 300 rpm, 150 °C and 7 g/min as the optimized process conditions. Theoretical and experimental analyses indicated that shifts in UV–Vis spectra at different ITZ concentrations were due to conformational changes in ITZ, which were confirmed through density functional theory (DFT) calculations and infrared spectroscopy. This work offers novel insights into the production and monitoring of ITZ-KVA64-ASDs, demonstrating that in-line UV–Vis spectroscopy is a powerful tool for real-time process monitoring and/or control.

## Graphical abstract

**Figure:**
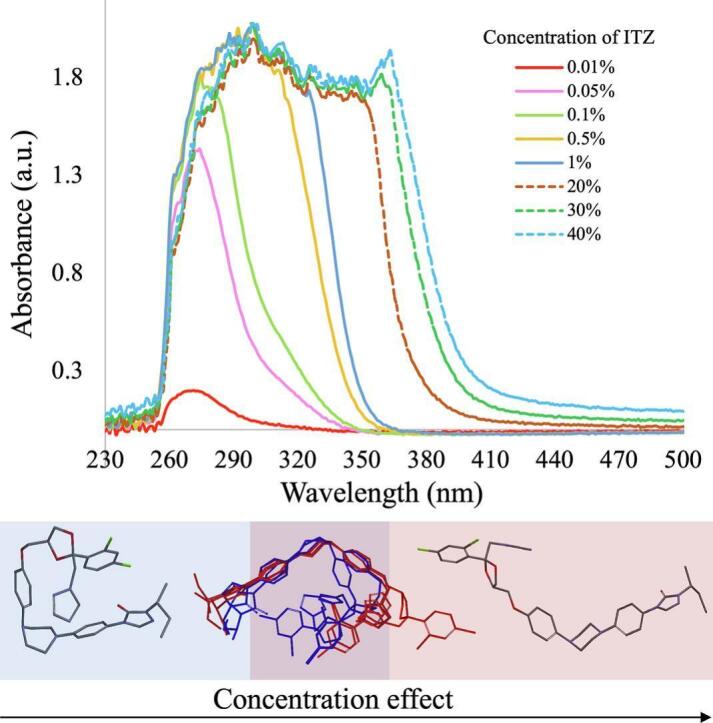

## 1 Introduction

Recent data indicates that approximately 40 % of marketed drugs and nearly 90 % of new chemical entities in development fall under Classes II and IV of the Biopharmaceutics Classification System (BCS). These classes are characterised by poor solubility and variable permeability: Class II drugs exhibit good permeability but poor solubility, while Class IV drugs have both poor solubility and poor permeability (Kalepu and Nekkanti, 2015; Bhalani et al., 2022; Andreas et al., 2018; Rosenberger et al., 2018).

To address the growing number of poorly soluble drugs, amorphous solid dispersions (ASDs) have gathered significant interest in recent decades (Pandi et al., 2020; Moseson et al., 2024). ASDs consist of the drug dispersed at the molecular level within a hydrophilic polymer matrix. In the amorphous phase, drug molecules are disordered, leading to an increased dissolution rate (Lin et al., 2018; Démuth et al., 2015). This improved dissolution is further enhanced by increased wettability from the hydrophilic carrier and reduced drug agglomeration (Feng et al., 2018). The amorphous state can enhance solubility up to 1600-fold compared to the crystalline form (Lakshman et al., 2008). However, amorphous solid dispersions (ASDs) face stability challenges due to their disordered molecular structure and the higher free energy associated with the amorphous state (Vasconcelos et al., 2016). While the amorphization itself does not prevent recrystallization, the resulting steric hindrance and non-covalent bonding between the drug and polymer can mitigate this process (Li et al., 2015; Crowley et al., 2007). These interactions create a miscible one-phase system with a negative Gibbs free energy, promoting stability (Agrawal et al., 2013). Key factors affecting ASD stability include the glass transition temperature (Tg), molecular mobility, drug-polymer miscibility, drug molecular weight, recrystallization temperature, and storage conditions (Lin et al., 2018).

Itraconazole (ITZ) was selected as the model drug for this study, representing an extreme case of a BCS Class II drug due to its bioavailability being limited by its dissolution rate in the gastrointestinal tract (Verreck et al., 2003). ITZ is highly permeable, with a logP of 5.66 at pH 8.11, but has low solubility, less than 1 ng/mL at neutral pH in aqueous solution (Miller et al., 2008). As a weakly basic drug, its solubility is pH-dependent, increasing in acidic conditions due to ionization (Dinunzio et al., 2012; Sarode et al., 2013). ITZ is a synthetic, triazole-based, lipophilic, and hydrophobic compound (Miller et al., 2008). Despite its polar rings, which theoretically should interact with water, ITZ's large molar mass (705.64 g/mol) and numerous non-polar C—H bonds contribute to its poor solubility (Reintjes, 2011).

Interestingly, ITZ also exhibits a unique morphology, forming multiple liquid crystal (LC) phases, particularly the thermotropic nematic and smectic A phases. These phases lie between crystalline and isotropic states, maintaining molecular order along specific axes (Atassi et al., 2013). The impact of this LC behavior, coupled with ITZ's highly anisotropic, rod-like structure (Mugheirbi and Tajber, 2015), on ASD formation has not been extensively studied, adding complexity to ITZ product formulation.

Various strategies have been explored to enhance ITZ's solubility, including co-crystals, cyclodextrin complexation, surfactant addition, mesoporous silica, self-emulsifying formulations, lipid nanoparticles, polymeric micelles, nanosuspensions, and solid dispersions (Anjum et al., 2024; Dhumal et al., 2024; Huang et al., 2022; Zhang et al., 2013). More advanced formulations include nanoparticles, ordered mesoporous silica, cyclodextrin nanosponges, laponite nanohybrids, and electrospun nanofibers (Engers et al., 2010).

Studies have shown that ITZ-polymer formulations, particularly those with Soluplus® (SOL), Kollidon® VA64 (KVA64), and HPMC, significantly increase ITZ solubility (Six et al., 2003b; Verreck et al., 2003; Zhang et al., 2013; Thiry et al., 2016; Davis et al., 2018). For example, Onmel®, a marketed ITZ melt extrusion product, contains 40 % ITZ and 60 % HPMC, offering a 2.3-fold increase in oral bioavailability compared to Sporanox® capsules (Repka et al., 2018).

Quality by Design (QbD) principles and data-driven models were used to track critical quality attributes (CQAs) in production. The integration of established PAT tools such as NIR, Raman, and UV–Vis spectroscopy in Hot Melt Extrusion (HME) has significantly improved pharmaceutical manufacturing efficiency, as documented in prior studies (e.g., Patel et al., 2023; Kumar and Singh, 2023; Almeida et al., 2020; Wesholowski et al., 2018; Schlindwein et al., 2018).

These tools allow real-time monitoring and immediate adjustments to critical process parameters, ensuring consistent product quality, reducing batch failures, and minimizing end-product testing. The integration of PAT directly into HME improves control over the extrudate's composition, reduces waste, boosts production speed, and lowers quality control costs. Additionally, PAT supports regulatory compliance by providing detailed process documentation and traceability, essential for pharmaceutical safety. This approach not only ensures regulatory compliance but also fosters innovation, making HME processes more efficient and cost-effective (Smith and Robinson, 2024).

This study focuses on the application of real-time in-line Process Analytical Technology (PAT) to monitor product quality during the manufacturing of amorphous solid dispersions (ASDs) and explores the effects of low ITZ concentrations on UV–Vis spectral changes. These findings are supplemented by density functional theory (DFT) simulations and infra-red spectroscopy analysis. Furthermore, we investigate the effects of shear forces during extrusion on UV–Vis spectral outcomes, revealing previously unreported conformational changes within the ITZ-KVA64 ASD system. This approach not only enhances the understanding of the formulation but also contributes to the development of more robust manufacturing processes in pharmaceutical production.

## 2 Materials and Methods

### 2.1 Materials

Itraconazole (ITZ), the active ingredient, was purchased from Wessex fine chemicals (Kent, UK). Kolllidon® VA64 (KVA64) was donated by BASF (Germany). Onset melting, degradation and glass transition temperatures for ITZ are 166.6 °C, 200–220 °C and 59–65 °C (amorphous ITZ) respectively. KVA64 has a glass transition of around 105 °C.

### 2.2 Methods

#### 2.2.1 Hot melt extrusion and in-line UV–Vis spectroscopy

Blending of the ITZ and polymers physical mixtures (PMs) was done using a V-cone mixer (Pharmatech, UK) for 20 min per 200 g batch. Nano-16 extruder (Somerville NJ, USA) with 16 mm co-rotating twin screws was used, with conveying (GF-A3–20-30, GF-A3–15-30 and GF-A3–10-30) and kneading (KB 7–3–15-60 F) elements. Three of the four heating zones were maintained at 120, 130, and 140 °C, while the fourth zone, known as the die zone, was adjusted between 140 and 160 °C across different extrusion experimental designs. Feed rates ranged from 7 to 9 g/min using the FW20 FlexWall gravimetric feeder (Brabender Technologie, Germany). Screw speeds were varied between 200 and 400 rpm. The HME process was continuously monitored using the in-line UV–Vis spectrophotometer (Inspectro X ColVis-Tec, Berlin, Germany) setup in transmission using optical fiber cables with two probes (TPMP, ColVisTec, Berlin, Germany). Transmittance data was collected from 230 to 816 nm with a resolution of 1 nm. Data collection frequency was 0.5 Hz, and each spectrum was taken as the average of 10 scans. The probes were positioned 0.85 mm apart, with an allowable variation of ±0.05 mm. The probe signal was optimized to be between 40,000 to 60,000 counts for each extrusion.

The extruder and UV–Vis spectrophotometer data were simultaneously collected using the PharmaMV software platform (Applied Materials USA). Steady-state calculations used transmittance values across 230–780 nm at 10 nm intervals. The PharmaMV platform displayed the maximum standard deviation within a 4-min window, and a threshold of 5 % standard deviation was used to define steady state. Our approach to detecting steady-state conditions relies on real-time data from the UV–Vis system rather than residence time estimates, ensuring direct confirmation of product stability.

The extrudates obtained were cooled to room temperature using a conveyor belt, pelletized (Accvapak System Ltd., UK) into 1 mm pellets and milled using a ball mill (Retsch, Germany) at 20 Hz for 10 min.

#### 2.2.2 Quality by Design Approach

The Quality Target Product Profile (QTPP) forms the foundation of product development and is the initial step in applying Quality by Design (QbD) principles (ICH, 2009; Yu et al., 2014). The QTPP proposed for the intermediate ITZ ASDs is shown in Table 1.

**Table 1:** Quality target product profile of ITZ-KVA64 ASD intermediate drug product (IDP).

Quality Attributes	Target
Intended use	Antifungal medication
API	Itraconazole
Drug system^a^	30 % itraconazole (ITZ) amorphous solid dispersion (ASD)
Stability	Amorphous itraconazole content for at least 6 months
Crystallinity	None
Appearance	Transparent extrudates
Content Uniformity	Meets Pharmacopeia acceptance criteria

a In-process (HME) quality material attributes.

Unlike the Quality Target Product Profile (QTPP), which focuses on the desired clinical outcomes, Critical Quality Attributes (CQAs) reflect the key characteristics of the final product and manufacturing process. For ITZ-KVA64 amorphous solid dispersions (ASDs), which in this case is an intermediate product, potential CQAs include content uniformity, crystallinity, solubility, and stability. Among these, crystallinity was identified as the most critical attribute for monitoring the hot melt extrusion (HME) process and was used as the primary response in the sequential experimental designs. The transparency of the ASDs is a direct indicator of the drug's crystallinity within the polymer matrix—opaque extrudates suggest drug recrystallization.

Identifying the Critical Process Parameters (CPPs) and Material Attributes (MAs) that influence CQAs is essential. In HME, upstream operations include blending and feeding, while downstream processes involve cooling, pelletizing, and milling. Potential CPPs for the extrusion process include screw speed, feed rate, barrel temperature, and screw configuration (Saerens et al., 2013; Censi et al., 2018). MAs are the properties of the drug and excipients that must remain within defined limits to ensure product quality. Examples of MAs in HME include the concentration, melting temperature, glass transition temperature, and degradation temperature of both the active pharmaceutical ingredient (API) and polymers (Evans et al., 2019).

It is critical to assess the main effects and interactions of CPPs and MAs to establish control over the CQAs (Van Buskirk et al., 2014). This can be achieved through risk assessment tools, such as those outlined in ICH Q9R1, 2023. For example, Almeida et al. (2020) conducted a Failure Mode and Effect Criticality Analysis (FMECA) of the HME process, utilizing the same in-line UV–Vis PAT tool. Readers are encouraged to refer to this publication for detailed information. It should be noted that, the quality of risk analysis depends more on the depth of knowledge about the product and process than on the specific tools or approaches used, making it somewhat subjective to the expertise of the team conducting the analysis.

A sequential Design of Experiments (DoE) approach (Politis et al., 2017) was adopted to obtain optimized processing conditions for producing ITZ ASDs (Table 2). The software used to produce DoEs and conduct statistical analysis was JMP® Pro 17.2 (JMP®, 2023). A fractional factorial design (screening design, DoE 1) was initially conducted to explore four factors: ITZ concentration (20 to 40 %*w*/w), die temperature (140 to 160 °C), screw speed (200 to 400 rpm), and feed rate (7 to 9 g/min). Eleven runs were performed for a 2 level, 4 factors fractional factorial with 3 centre points. The results from this set of experiments led to the second design (central composite design, DoE 2) that explored the interaction between the feed rate (5, 7 and 9 g/min) and screw speed (200, 300 and 400 rpm). The concentration (30 %) and temperature (150 °C) were kept constant (see results and discussion section). Lower feed rate was explored due to poor powder flow properties of the mixture. This also enabled results from both designs to be compared. Each sample was prepared as a separate batch and fed sequentially, with the sequence blocked by scan rate to minimize waste caused by feeder recalibration between experiments. The extruder operated continuously throughout the processing of all samples. Steady-state conditions between experiments were confirmed as previously described. This design (DoE2) included two center points, but additional samples, prepared under identical conditions, were used for the subsequent verification and validation experiments which provided assurance of the reproducibility of the process conditions and the robustness of the formulation.

**Table 2:** Fractional factorial screening experimental design (DoE 1) showing the 11 runs for the 4 parameters investigated (temperature, feed rate, concentration and screw speed) with the addition of 3 center points. Center composite design (DoE 2) for optimization of 2 factors (screw speed and feed rate) with 2 center points.

Fractional Factorial - Screening Design – DoE 1				
Run	ITZ (%)	Temperature (°C)	Screw Speed (rpm)	Feed Rate (g/min)
1	20	140	200	7
2	20	140	400	9
3	20	160	200	9
4	20	160	400	7
5	30	150	300	8
6	30	150	300	8
7	30	150	300	8
8	40	140	200	9
9	40	140	400	7
10	40	160	200	7
11	40	160	400	9

**Center Composite Design – DoE 2**				
1	30	150	400	5
2	30	150	200	5
3	30	150	300	5
4	30	150	300	7
5	30	150	300	7
6	30	150	200	7
7	30	150	400	7
8	30	150	400	9
9	30	150	300	9
10	30	150	200	9

The final step to the sequential design approach was to conduct a verification study. This involved running four random points within the design space (DS) obtained, e.g., 300 rpm at 7 g/min, 300 rpm at 6 g/min, 380 rpm at 7.5 g/min and 360 rpm at 8.5 g/min. A final set of extrusion experiments (validation) were conducted of the optimized processing conditions (30 % ITZ, 150 °C, 300 rpm and 7 g/min) by running the experiment continuously for 3 h to confirm process stability during steady state processing over three different days between 4 and 6-month.

## 3 Characterisation of samples

### 3.1 Off-line techniques

Differential Scanning Calorimetry (DSC) experiments were carried out using the Polyma 202 (Netzsch, DE) DTA and analysed using the thermal analysis software Proteus® 80 software (Netzsch, DE). The DSC was calibrated with pure indium initially. The scan rate was 10 °C/min for the heating runs. All runs were conducted under a purge of nitrogen gas (20–40 mL/min). For each scan, samples of 5 mg were weighed and placed in an aluminum pan.

The equipment D2 Phaser X-Ray diffractometer (Bruker, DE) was used to analyse physical mixtures (PM) and extrudate samples. Powder samples were manually dispersed into a circular metal sample holder using a glass slide with hand pressure to obtain levelled surface. Samples were subject to measurements from 4.0 to 40.0 2θ angles at step time 0.5 s and step size 0.02–0.006 2θ, depending on the sample.

The Alpha Platinum FTIR spectrophotometer (Bruker, DE) was operated by the software OPUS 7.5 (Bruker, DE) using transmission mode. 16 infra-red spectra were averaged in the range of 400–5000 cm^−1^ with resolution of 2 cm^−1^. The reference materials and samples were dispersed into spectroscopic potassium bromide (KBr) for transmission measurement.

Off-line UV–Vis spectroscopy was performed using the same spectrophotometer for the in-line measurements (section 3.2) but using a holder for solid discs produce using vacuum compression molding (MeltPrep, 2024).

### 3.2 In-line UV–Vis spectroscopy data collection and analysis

Several data acquisition steps were conducted, which involved selection of the steady state UV–Vis transmittance signal, pre-processing to convert the transmittance data into absorbance and multivariate analysis (MVA) using the JMP® Pro software. The CIELab System (ASTM, 2022) was used to measure the colour of the samples. L* value of 100 indicates transparent samples which was desirable. Transmittance spectra from 230 to 816 nm in the UV–Vis region with a resolution of 1 nm, were collected. Steady state transmittance data was normalized by correcting the baseline using the spectrum of the pure polymer in its molten state at the start of each experimental day. This was conducted by calculating the average absorbance at each wavelength for KVA64 and subtracted from the samples' spectra. The normalization step allows variability between the experimental days to be reduced and allows comparison of spectra across different extrusions. This will remove any baseline shift that might occur due to any unmeasured disturbances that might affect the process or the UV–Vis on a different experimental day.

### 3.3 Itraconazole molecular simulations

The conformational analysis and geometry optimization of itraconazole were carried out using Spartan 20 (Wavefunction Inc., Irvine CA, USA). Starting from a three-dimensional model, a conformational search employed the Merck Molecular Force Field (MMFF94), followed by geometry refinement with the Parametric Method 6 (PM6) and Density Functional Theory (DFT). Optimization used the B3LYP/6-31G* level in the gas phase. Time-Dependent DFT (TD-DFT) was then applied to the optimized rotamers to assess their behavior in the excited state.

## 4 Results and discussions

### 4.1 Fractional factorial screening design (DoE 1)

#### 4.1.1 In-line UV–Vis spectroscopy

**Fig. 1:**
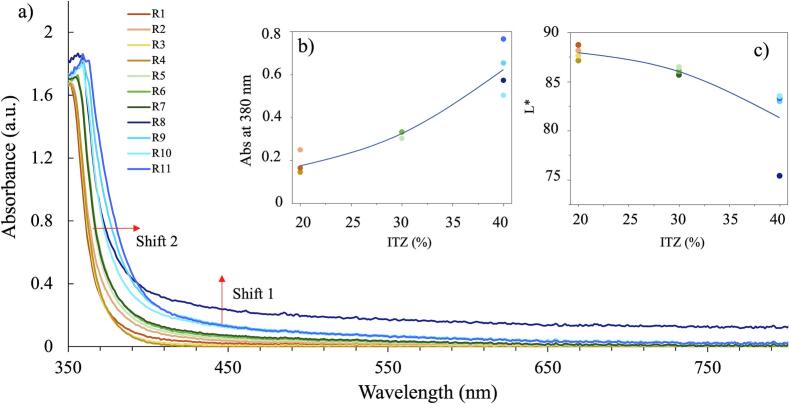
a) Absorbance spectra for 11 runs of DoE 1: b) Baseline shift 1 for absorption values at 380 nm as a function of concentration of ITZ, c) L* values as a function of concentration of ITZ.

Absorbance spectra for the 11 runs are shown in Fig. 1a. Two main changes were observed; a shift to a higher wavelength (360 to 420 nm) and a shift in absorbance at the baseline in the visible range (0.19 to 0.29). The shift in the UV range can be attributed to the increase in ITZ concentration from 20 to 40 % (Fig. 1a shift 2) which has also been reported by Wesholowski et al. (2018) for carbamazepine and theophylline and Schlindwein et al. (2018) for piroxicam. This could be attributed to different levels of molecular interactions between the ITZ and KVA64 leading to the formation of structures with probably different conformations. ITZ is highly susceptible to this as it is an anisotropic API with the ability to form different liquid crystal phases (Kozyra et al., 2018). The shift observed may be attributed to the unique liquid crystal behavior of ITZ, but the exact reason for this shift has not yet been reported. This shift in the UV range is visible at both high and low concentration ranges (Fig. 1b).

Shifts in the baseline (Fig. 1a, shift 1) usually occur due to the presence of bubbles or scattering (due to oversaturation and/or phase separation), previously reported by Schlindwein et al. (2018). These conditions were most likely to fail target quality as at high concentrations, low temperature, low screw speed and high feed rate caused oversaturation of the system with insufficient mixing of the ITZ and KVA64. This agrees with the values reported for L*, as the concentration increases, L* decreases (Fig. 1c).

#### 4.1.2 Selection of absorbance value output

**Fig. 2:**
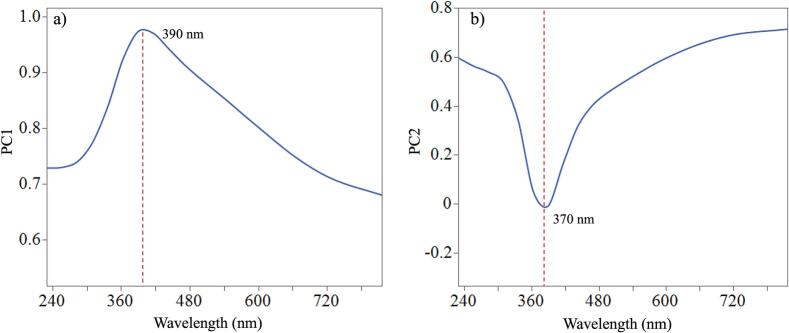
PC1 (a) and PC2 (b) loadings for DoE 1.

Using principal component analysis (PCA) loading plots, the selection of the wavelength range demonstrating the greater signal changes was obtained. This allows the selection of the wavelengths for analysis which has the maximum contribution to changes (Almeida et al., 2020). PC1 and PC2 were applied to the entire spectra and the loadings obtained are shown in Fig. 2a and Fig. 2b respectively.

**Fig. 3:**
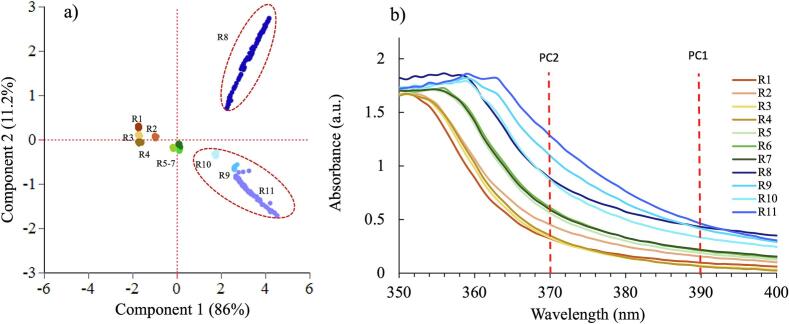
a) Score plot of the PCA, b) UV-Vis spectra for DoE 1. Four factors investigated (ITZ %, Temperature, Screw speed and Feed rate).

The score plot (Fig. 3a) shows scatter of the runs related to the first and second PCs. PC1 explained 86 % of the variation and can be attributed to the shift in the absorbance of runs 8 to 11 at 390 nm. PC2 explained 11.2 % of the variation and can be attributed to the increase in absorbance at 370 nm for the sample runs, with the biggest increase for sample R11. Along the PC2 (y-axis) the runs appear to be in clusters according to the ITZ concentration e.g., brown lines represent 20 % ITZ, green 30 % ITZ and blue 40 % ITZ. The absorbance (Abs) at 370 and 390 nm were therefore modelled as CQA responses (Fig. 3b).

#### 4.1.3 Modelling the output CQA responses

**Fig. 4:**
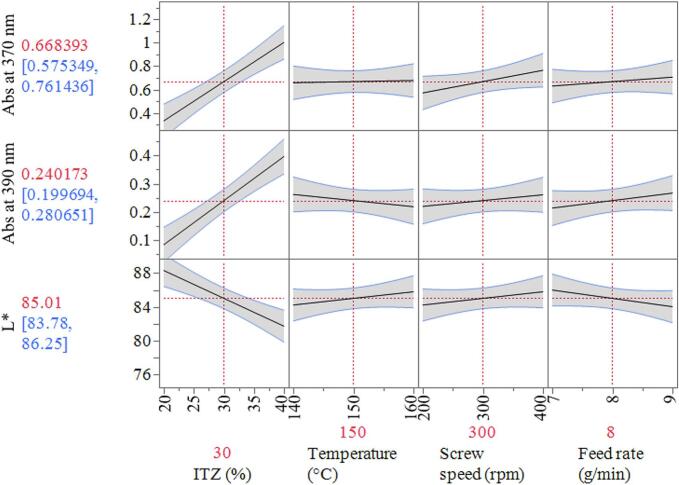
Prediction profiler plots showing the relationship between the 3 responses (L*, Abs at 390 and 370 nm) and 4 factors investigated (ITZ %, Temperature, Screw speed and Feed rate).

For each response the main effects and two factor interactions were modelled (Fig. 4). A limit of 83 was set as a minimum target for transparency. L* remained mostly above 83 for ten out of the eleven samples and the extrudates were all transparent in appearance, so passed the L* criterion. Sample R8 was translucent and exhibited the lowest L* of 75.4, which failed to meet the CQA limit for L*. The high feed rate (9 g/min) affects more (RTD), which can lead to insufficient mixing and inadequate time for solubilization. The increased feed rate can result in a short RTD, as materials spend less time in the extruder. This can adversely affect the mixing and solution process, leading to suboptimal interactions between ITZ and KVA64. Consequently, these factors can impact the overall effectiveness of the amorphous solid dispersions.

**Fig. 5:**
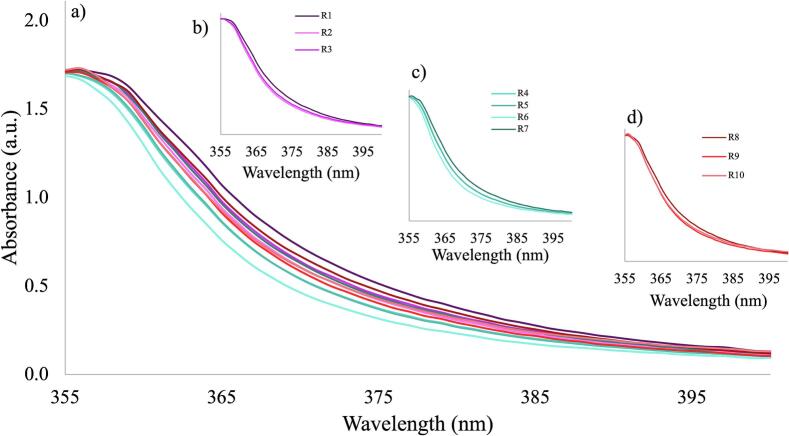
Absorbance results for DoE 2: a) Full Spectra with the 10 samples, b) runs at 5 g/min, c) runs at 7 g/min and d) runs at 9 g/min.

This can lead to insufficient mixing to the ITZ and KVA64, hence causing a shift in absorbance. The prediction profiles shown in Fig. 5c indicate that, within the parameter ranges investigated, only the concentration of ITZ has a significant impact on the responses evaluated (absorbance at 370 nm, 390 nm and wavelength at absorbance 1).

Overall, an adequate model could be built from this data, however ITZ concentration % is the only factor that shows an “detectable” effect in the CQA responses analysed. A summary of the statistical analysis for the 3 responses analysed (DoE 1) is available as supplementary material (**Table S1**).

#### 4.1.4 Differential scanning calorimetry and X-Ray diffraction for DoE 1

DSC thermograms for all samples in the second heating step are shown in **Fig. S1a**. All samples, including sample R8 exhibited a one phase miscible system as there was an absence of the ITZ melting onset Tm at 166.63 °C. Piccinni et al. (2016) and Six et al. (2003a) also successfully produced ASDs of ITZ and KVA64 using HME ranging from 10 to 80 %, with a single Tg. The glass transition temperature (Tg) decreases as the drug concentration increases, ranging from 99 to 89 °C. The experimental Tg values were 56 °C for ITZ and 105 °C for KVA64. Since Tg reflects chain mobility, this suggests a potential interaction between the drug and polymer (Li et al., 2013). At higher drug concentrations, the polymer may experience a plasticizing effect due to hydrogen bonding interactions (Amharar et al., 2014). Similar observations were made by Six et al. (2003b), who reported that ITZ-HPMC ASDs produced via hot melt extrusion (HME) showed a lower Tg with increased ITZ concentration.

The X-Ray diffractograms of the DoE 1 samples are shown in **Fig. S1b**. Crystalline ITZ exhibits characteristic peaks at 2θ equal to 11.75°, 17.80°, 18.94°, 21.84° and 27.84°. These were also reported by Feng et al. (2018). The peaks also correlate to ITZ polymorph form I peaks reported by Zhang et al. (2019). There was an absence of the characteristic crystalline peaks of ITZ in all ASD samples. The diffraction patterns of the samples also exhibit a similar halo pattern to pure KVA64, which is amorphous. High molecular weight polymers, such as KVA64, lack a specific pattern so do not have defined crystals patterns (Thakral et al., 2016). In a study conducted by Piccinni et al. (2016), 30, 40 and 50 % ITZ and KVA64 ASDs were produced by HME. They also demonstrated the absence of characteristic ITZ peaks. However, the PXRD technique is limited to detecting crystallinity below 10 % (Crowley et al., 2007).

Overall, for all samples produced using HME (high and low concentrations), the results showed that all the extrudates were amorphous, regardless of the different screw speeds, feeding rates and die temperature ranges used. UV–Vis spectroscopy, in the visible range, has been shown to be more sensitive in detecting amorphous states compared to differential scanning calorimetry (DSC) or X-ray diffraction, as demonstrated in previous studies (Wesholowski et al., 2018; Schlindwein et al., 2018). In our study, this enhanced sensitivity allowed us to confidently assert that extrudates produced through hot melt extrusion (HME), that meets the required QTPP, remained in an amorphous state. This capability of UV–Vis supports its application as a reliable method for characterizing amorphous solid dispersions.

### 4.2 Process optimization (DoE 2)

Due to concentration being the dominant parameter, the influence of screw speed and feed rate were not observed in DoE 1 experiments which showed an almost flat response values as a function of the parameters investigated (Fig. 4). Therefore, further optimization of these CPPs was considered. A central composite design was adopted to explore the interaction between the feed rate (5 to 9 g/min) and screw speed (200 to 400 rpm). The concentration (30 %) and temperature (150 °C) were kept constant. The working design space was then proposed.

#### 4.2.1 In-line UV–Vis spectroscopy

UV–Vis spectra for DoE 2 samples are shown in Fig. 5a. Shifts in the visible range were seen at constant feed rate and different screw speeds. For example, at constant feed rates of 5 (Fig. 5b), 7 (Fig. 5c), and 9 (Fig. 5d) g/min there is a shift to higher wavelength in the UV region for runs R1, R7 and R8 respectively. There is no evidence for shifts in visible range in this DoE. Samples from runs 1 to 9 were transparent in appearance. However, it was not possible to continually collect samples from R10 due to high feed rate (9 g/min) and slow screw speed (200 rpm) of the process conditions. This caused feeding zone 1 to overflow and the extruder to clog. Collection of in-line UV–Vis data was possible only at the beginning of the run.

#### 4.2.2 Selection of absorbance value output

**Fig. 6:**
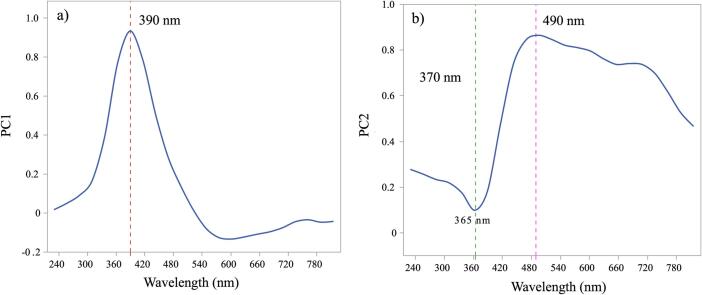
PC1 (a) and PC2 (b) loadings for DoE 2.

**Fig. 7:**
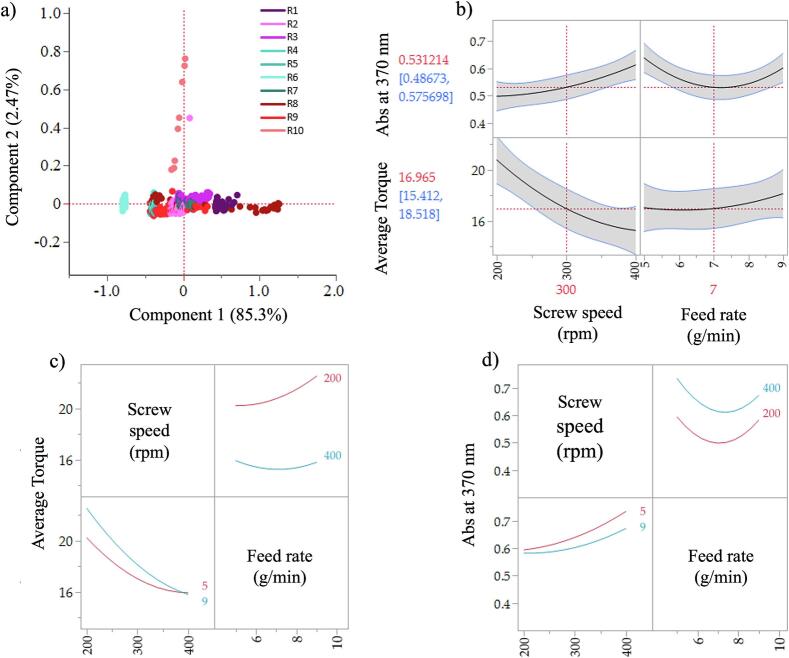
Plots for DoE 2. a) Score plot of the PCA. b) Prediction profiler showing the effect of screw speed and feed rate on the responses average torque and absorption at 370 nm. Interaction profile of the factors with respect to c) the average torque response and d) absorption at 370 nm responses.

Three wavelengths were found which were impacted by the CPPs the most: 390 nm in PC1, 365 and 490 nm in PC2 (Fig. 6**)**. The PC results were found to be very similar to those found in DoE 1. The score plot in Fig. 7a shows that PC1 accounted for 85.3 % of the variation while PC2 accounted for 2.47 % of the variation. The effect of the screw speed can be attributed to the PC1 region. R1 and R8 samples can be explained using PC1 and can be associated with high screw speed of 400 rpm. PC2 shows variabilities within the spectra collected for R10. This was reflected during the HME as collection of steady state samples for R10 was not possible due to the high feed rate (9 g/min) and low screw speed of 200 rpm which caused jamming in the feeding zone after a period. Based on the PC1 results modelling was conducted on Abs value of 390 nm along with modelling at 370 nm for statistical comparison with DoE 1 samples.

The statistical analysis of the responses is available as supplementary material (**Table S2**). The R^2^ adjusted for the absorbance at 370 nm was higher compared to absorbance results at 390 nm: 86.5 and 75.7 % respectively. Along with the F ratio explaining 12.5 % of the variation compared to 6.6 %. The model for absorbance at 370 nm demonstrated higher statistical significance as *p* values were less than 0.05 for the terms screw speed and feed rate square (quadratic term). An increase in absorbance can be seen with the increasing screw speed.

For the L* response the R^2^ adjusted was low (45), with a small signal to noise F ratio (2.47). Neither the screw speed nor feed rate had an impact on L*. Sorted parameter estimates for the L* could not be calculated due to all the L* readings being around 84, which was expected for concentration of 30 % ITZ (all samples met the QTPP for L*). An alternative response associated with the process (torque) was considered and modelled to obtain a potential design space.

Torque was selected as a key process-related response because it serves as an important indicator of the impact of changing operational conditions within the extruder. While we acknowledge that residence time is critical for understanding phase states and potential crystal residues, torque can provide insights into the mixing efficiency and overall energy input during the extrusion process.

Monitoring torque allows us to define operational limits relative to feed rate and screw speed, which are essential for optimizing the extrusion process. A stable torque reading can indicate adequate mixing and consistent processing conditions, which can indirectly influence residence time and, consequently, the phase behavior of the formulation.

Additionally, by evaluating both responses, Abs at 370 nm and torque together, we can better understand the impact of critical process parameters on the quality of the final product. The prediction profiler and interaction profiles are showing in Fig. 7b-d.

### 4.3 Design space, process verification and validation

**Fig. 8:**
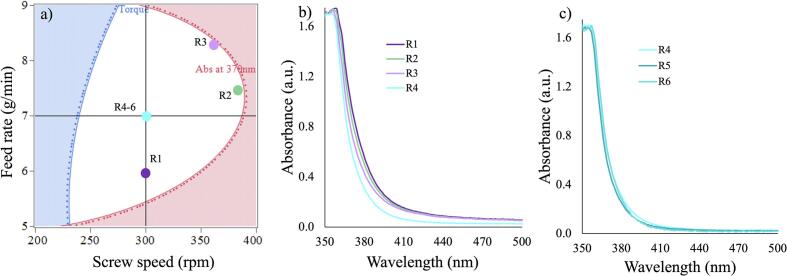
a) Design space obtained by modelling the abs at 370 nm and the average torque values for each run; verification points marked in the white borderline areas within the DS region (300 rpm and 7 g/min, 300 rpm and 6 g/min, 380 rpm and 7.5 g/min and 360 rpm and 8.5 g/min). b) UV-Vis spectra for the 4 verification samples. c) Validation runs performed at 3 different days within 4 to 6-month interval.

The design space generated using DoE 2 results is shown in Fig. 8a. The higher limit for the absorption at 370 nm was set to 0.9 based on the results for DoE 2. The maximum limit for the torque was set to a value of 19 N.m to make ensure continuous production performance. The contour lines and shaded regions for the average torque (blue) and Abs at 370 nm (red) show the boundary to achieve ASDs which meet the quality target product profile. Any combination of the screw speed and feed rate selected within the boundary (working DS, white region) will produce 30 % ITZ-KVA64-ASDs with the desired quality attributes and process performance. On the contrary, outside of this region any ITZ-KVA64-ASDs produced are likely to fail the quality target criteria. Verification of this DS was conducted by selecting four points within the DS at random; 300 rpm and 7 g/min, 300 rpm and 6 g/min, 380 rpm and 7.5 g/min and 360 rpm and 8.5 g/min as indicated in Fig. 8b.

All runs within the design space yielded amorphous samples. Based on the results in Fig. 8b, the optimal processing conditions for the final ITZ ASD were determined to be 30 % ITZ, 150 °C, 300 rpm, and 7 g/min. Verification runs confirmed the formation of amorphous samples, showing a single Tg (**Fig. S2a**) and no crystalline ITZ peaks (**Fig. S2b**). The Tg values ranged from 94.4 to 96.4 °C. To validate the optimized processing conditions, the experiment was repeated over three hours on three different days over a period of 4 to 6 months between them (R4, R5, and R6 in Fig. 8c). The stability of UV–Vis spectra, along with DSC and XRD characterizations, further evidenced the amorphous nature of the extrudates.

### 4.4 Investigation of conformational changes in itraconazole ASD

This work also focuses on attempting to address the changes in conformational morphology of ITZ within the ITZ-KVA64-ASD system contributing to the shift observed in the in-line UV–Vis results during HME process. First, the effect of lower ITZ concentration on the in-line UV–Vis spectra was investigated, second simulations were used to conduct theoretical density functional theory calculations (DFT) of ITZ to understand the conformation and its potential interactions with KVA64 in the ASD and finally determining whether the shearing induced by the extrusion could potentially contribute to shift in the UV–Vis spectra. The simulations of ITZ were carried out using the software Spartan 20.

#### 4.4.1 In-line UV–Vis spectroscopy of low concentration ITZ ASD

**Fig. 9:**
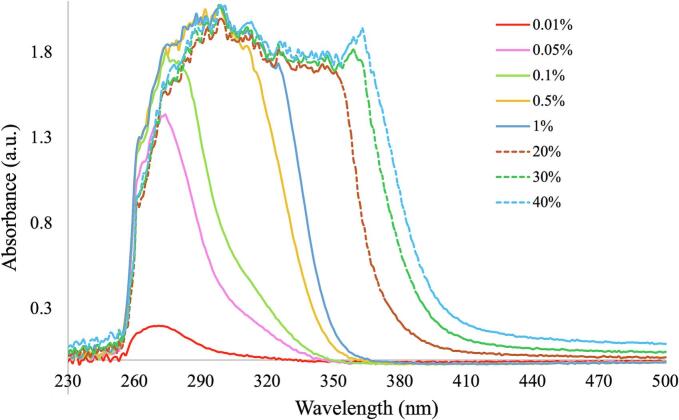
In-line UV-Vis results for the low (full lines) and high (dotted lines, from DoE 1) concentration ITZ-KVA64-ASDs.

Univariate HME experiments were conducted to see the effect of low ITZ concentrations on the in-line UV–Vis spectra (0.01, 0.05, 0.1, 0.5 and 1 % *w*/w) when producing ITZ-KVA64-ASDs. These were produced at 150 °C, 300 rpm screw speed and feed rate of 7 g/min, based on the optimized HME conditions described before. The in-line UV–Vis spectra for the low concentrations, along with run 1 (20 % ITZ-KVA64-ASD produced at 140 °C, 300 rpm and 7 g/min), run 6 (30 % ITZ-KVA64-ASD produced at 150 °C, 300 rpm and 8 g/min) and run 9 (40 % ITZ-KVA64-ASD produced at 140 °C, 400 rpm and 7 g/min) from DoE 1 are shown in Fig. 9. The three DoE 1 runs were plotted for the purpose of comparison of spectral shifts at higher ITZ concentrations.

The results show an increase in the shift in the UV region from 290 to 420 nm from 0.01. to 40 % ITZ. Comparing the shifts at the higher concentrations (R1, R6 and R9 from DoE 1), the transition in the UV range becomes more prominent. This shows that the shift in the UV range can be attributed to ITZ and is evident even at very low concentrations. A high concentration of the drug may cause saturation of the system, leading to a decrease in transmission of the light and increase in absorbance (Bakeev, 2010), so lower concentrations were used for this study. Larger shifts were also observed between 0.01 and 0.05 % and 0.1 to 0.5 %.

#### 4.4.2 Statistical analysis of the in-line UV–Vis results

**Fig. 10:**
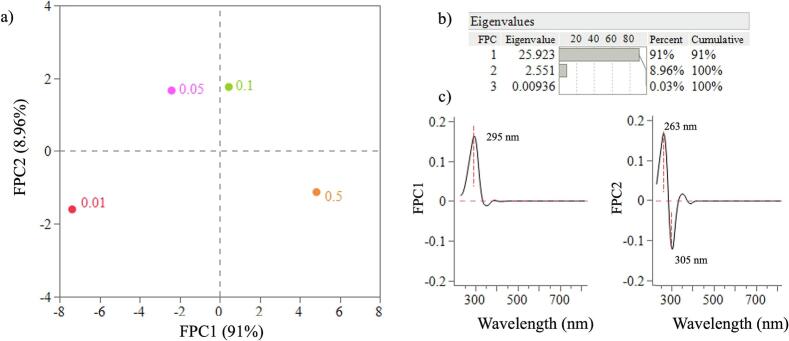
a) Score plot for the low concentration samples (0.01 to 1 %) showing the FPC1 versus FPC2 scores, b) Eigenvalues for the FPCA, c) FPC1 and FPC2 versus wavelength.

Using functional principal component analysis (FPCA), the low concentration range samples were evaluated. A B-spline quadratic model was applied. Through FPCA the effect of ITZ concentration on the absorbance as a function of wavelength can be understood more clearly and is easier to visualise using the score plot (Fig. 10a). The eigenvalue for the FPC1 was 25.923 which accounts for 91 % of the variation, with FPC2 accounting for 8.96 % of the variation (Fig. 10b). The FPC1 plot shows a peak at 295 nm and FPC2 shows two peaks at 263 and 305 nm (Fig. 10c). The FPC1 peak at 295 nm shows the biggest shift occurring from 0.01 % to 1 % ITZ concentration. This is also explained by the score plot where the difference between 0.01 % and the two higher concentrations, 0.5 and 1 % can be explained using FPC1, along the x-axis.

The first FPC2 peaks at 263 nm was due to the shift in UV range to a higher wavelength from the lowest ITZ concentration of 0.01 to 0.05 %. The second FPC2 peak at 305 nm, explains the shift from 0.01 % to 0.1 % ITZ. These shifts were observed as the less significant changes in the UV–Vis spectra as it only accounted for 8.96 % of the variation. This can also be observed from the score plot as the distance between the point at 0.01 % to 0.05 % and 0.1 % is greatest in the y-axis which is explained using FPC2. The score plot is also showing that the spectra for 0.05 % and 0.1 % ITZ are similar.

FPCA analysis indicated that the biggest changes in spectra are occurring between 230 and 400 nm range. As the concentration of ITZ increases this becomes more evident. To determine the conformations of ITZ which contribute to this difference in absorbance simulations of ITZ rotamers was conducted.

#### 4.4.3 Geometry optimization and UV–Vis absorption analysis of itraconazole

**Fig. 11:**
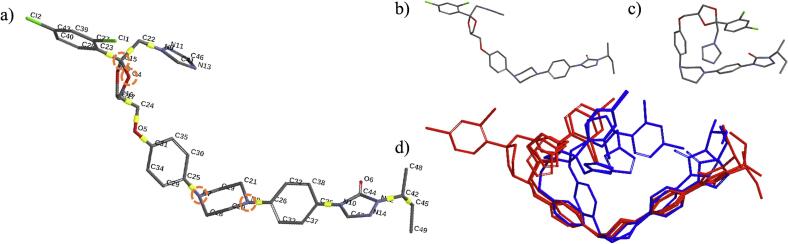
a) ITZ molecules with torsion points (highlighted in yellow and orange dotted circles) where rotamers were obtained, b) rotamer M0373, c) rotamer M0019, and d) alignment of six low-energy rotamers generated as listed in **Table S3**. (For interpretation of the references to colour in this figure legend, the reader is referred to the web version of this article.)

The geometry optimization of itraconazole (ITZ) rotamers and their impact on UV–Vis spectral properties were analysed using Density Functional Theory (DFT) and Time-Dependent DFT (TD-DFT), providing accurate spectral computations in alignment with previous studies (Corrêa et al., 2012; Santos et al., 2018; Gomes et al., 2019). First, 15 local minima rotamers were generated using the MMFF force field and subsequently refined through PM6 semiempirical geometry optimization (**Table S3**). Among these, the six lowest-energy rotamers (highlighted in bold in **Table S3**) were selected for detailed UV–Vis absorption analysis, showcasing their distinct absorption characteristics. Key torsion angles, especially around dihedral C36–C26–C25–C31, illustrated the structural flexibility of ITZ (Fig. 11a, **Table S3**). Conformer M0373 (Fig. 11b), featuring a near-planar structure, showed a redshifted absorption peak at 346.42 nm, whereas the more coiled M0019 (Fig. 11c) displayed a blue shifted absorption peak at 324.56 nm. Fig. 11d shows alignment of six low-energy rotamers generated as listed in **Table S3**. In a study by Kujawski et al. (2017), DFT calculations using the B3LYP/6-31G(d,p) approach in the gas phase identified a low-energy ITZ conformer with an absorption peak at 348.16 nm, closely matching the redshifted profile of rotamer M0373 in our study. Structural flexibility in ITZ significantly affects its spectral properties, where planar conformations correlate with redshifted and coiled structures with blue shifted absorption, highlighting how structural variations influence spectral response and behavior in different environments.

#### 4.4.4 FTIR spectroscopy of ITZ and calculated IR frequencies of ITZ rotamers

**Fig. 12:**
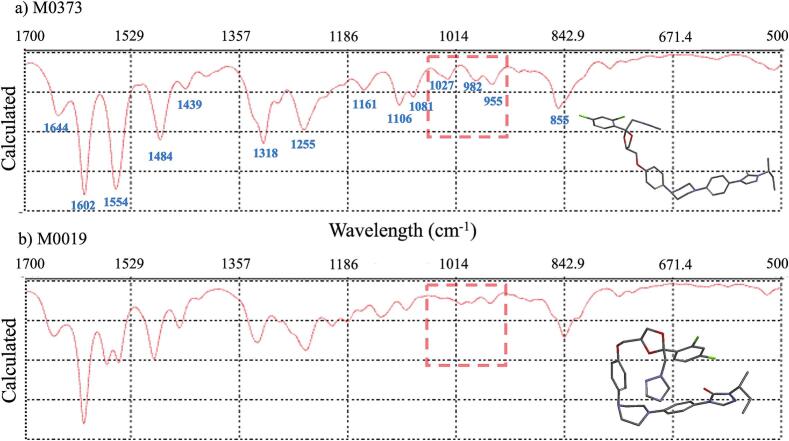
Infrared spectra generated for the minimum energy (a) M0373 and (b) M0019 rotamers.

**Fig. 13:**
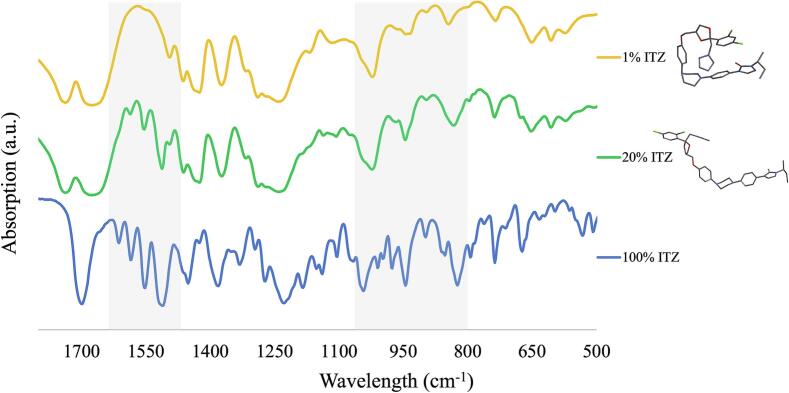
FTIR spectra of 100 % pure ITZ, 20 % ITZ-KOL-ASD and 1 % ITZ-KOL-ASD. The shaded areas show the differences between high (20 %) and low (1 %) concentrations compared to 100 % ITZ.

The characteristic FTIR absorption peaks of pure ITZ have been well documented in the literature (Kujawski et al., 2017; Feng et al., 2018). Comparative analysis of calculated IR spectra between M0019 and M0373 highlighted distinct absorption patterns, especially in the fingerprint region. Fig. 12 presents the IR spectra of both rotamers, where the peak at 1602 cm^−1^ for rotamer M0373 and 1603 cm^−1^ for M0019 is close to the experimental peak at 1608 cm^−1^ (Fig. 13), characteristic of the C=C stretching from the phenyl rings. Additionally, the peak at 1554 cm^−1^ for M0373 aligns with the experimental peak at 1588 cm^−1^ (Fig. 13). In the twisted rotamer M0019 (Fig. 12b), this peak splits into two at 1567 and 1547 cm^−1^ due to altered conformation of the phenyl rings, disrupting planarity and symmetry. M0373 also displays peaks at 1027 and 982 cm^−1^ (Fig. 12a), which correlate with the experimental peaks ranging from 1043 to 943 cm^−1^ (Fig. 13), indicative of C—O stretching from the cyclic ether group. In contrast, M0019 does not show peaks in this region (Fig. 12b).

FTIR spectra for crystalline itraconazole (ITZ) and high (20 %) and low (1 %) concentrations of ITZ-KVA64 ASD were obtained experimentally (Fig. 13) and compared to the calculated IR spectra for rotamers M0019 and M0373 using Spartan 20. (Fig. 12).

The lower intensity of C—O peaks in the 1072–977 cm^−1^ region suggests the presence of the M0019 rotamer, which absorbs at a lower wavelength (324.56 nm) in the UV–Vis spectrum. Conversely, the presence of C—O peaks indicates rotamer M0373, aligning with its higher UV–Vis absorption wavelength (346.42 nm). It can be seen from Fig. 13 that experimental data for low concentration (1 %) ITS ASD shows similar behavior as for the M0019, bending rotamer. At higher concentration (20 %) ITZ ASD the similarity to the M0373 rotamer is more likely, which exhibits the lowest energy in the DFT calculation and absorb at higher wavelengths in the UV–Vis. This provides evidence of different conformational changes of ITZ with the increase in concentration where the ITZ molecule is more aligned.

### 4.5 Impact of shear stress on ITZ conformation

**Fig. 14:**
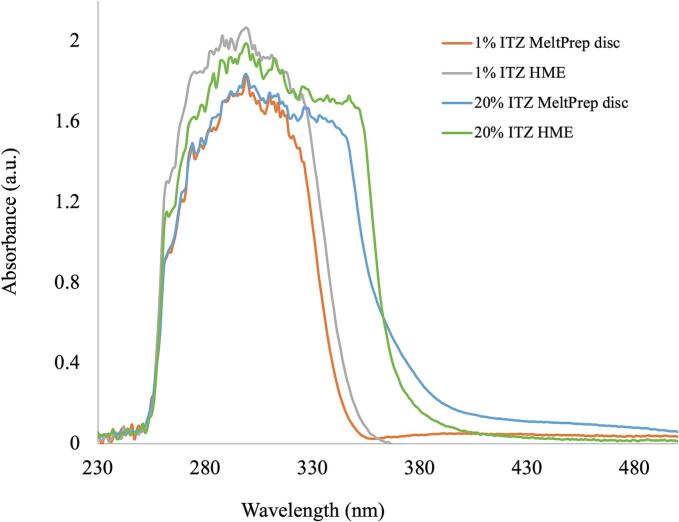
In-line and at-line UV-Vis spectra for 1 % and 20 % ITZ-KVA64-ASDs prepared with shear (HME) and without shear (vacuum compression molding – MeltPrep disc).

The final part of this work addresses whether shear during HME process from the co-rotating twin screws may have an impact on conformational changes exhibited by ITZ at low (1 %) and high (20 %) concentration. The shearing force plays a vital role in the molecular mixing of the drug into the polymer (Just et al., 2013; Solanki et al., 2019). To determine whether shear is a contributor to the shift seen in the UV–Vis region, discs of 1 % and 20 % ITZ-KVA64 were compressed at set temperatures of 150 and 160 °C respectively under vacuum, in static conditions using vacuum compression molding technique (MeltPrep, 2024). The discs were measured using at-line UV–Vis method with the same spectrophotometer used for the in-line experiments. The at-line spectra were compared to the corresponding in-line spectra. Fig. 14 shows that there is no significant difference between the spectra of the same concentration. However, a shift is observed to a higher wavelength from 1 to 20 % ITZ concentration even under static conditions (MeltPrep discs). This suggests that shear from the extrusion was not the reason for the shift in UV as the concentration of ITZ increases.

## 5 Conclusions

This paper provides a robust investigation of the manufacture of ITZ ASD using HME and in-line process technology with a systematic approach underpinned by QbD methods. A sequential DoE approach was used to manufacture and optimize ITZ-KVA64-ASDs using HME process. The manufacture process was continuously monitored using an in-line UV–Vis spectroscopy with probes designed for HME.

A fractional factorial screening design was adopted (DoE 1) to screen critical process and formulation parameters. Four factors were investigated, the ITZ concentration (20 to 40 %), die temperature (140 to 160 °C), screw speed (200 to 400 rpm) and feed rate (7 to 9 g/min). Out of the eleven samples produced, ten samples passed the target criteria which had an L* above 83 %. In-line UV–Vis spectroscopy results for DoE 1 showed two shifts in the absorbance spectra as the concentration increased. The first shift was clearly identified by the increase in wavelength values in the UV region from 369 to 375 nm at absorbance of 1. The second shift was at the baseline, in the visible region, which shifted the spectra to higher absorbance values. This was due to insufficient mixing of the API and polymer at high feed rate (9 g/min) and low screw speed (200 rpm) and/or the presence of bubbles causing scattering of light. The baseline shift in the visible range has been demonstrated in earlier publications as in some cases more sensitive than DSC and XRD, this is an important finding in this work. After screening of the four factors, it was evident from the prediction and interaction profiles that the ITZ concentration effect was dominant and was masking the effects of the other CPPs.

Therefore, for DoE 2, a central composite design was adopted to explore the interaction between the feed rate (5 to 9 g/min) and screw speed (200 to 400 rpm) only. The concentration (30 %) and temperature (150 °C) were kept constant. Ten samples were extruded, of which nine passed and one failed (R10 due to clogging of feeding zone). R10 sample, which failed, was extruded at 9 g/min with a low screw speed of 200 rpm as this caused overflow of the feeding zone so only a small sample size was collected. All L* values for the 10 samples were close to 84 and this response could not be used to in the model. An alternative response associated to the process, the torque, was evaluated as a function of the screw speed and feed rate. The responses, absorbance at 370 nm and torque were then used to develop a design space. All samples met the QTPP criteria for L* of minimum 83.

In the final step of the sequential DoE approach a validation study was conducted on the midpoint condition from the design space (30 %, 150 °C, 300 rpm, 7 g/min). HMEs were conducted over three different days over a period from 4 to 6-month to validate the optimized conditions to produce ITZ-KVA64-ASDs. The in-line UV–Vis spectra were stable across the three different days and the samples were quantified giving a final concentration of 30 ± 2 %. This enabled continuous monitoring of CPPs and multivariate analysis without extensive manipulation of the data. In-line UV–Vis spectroscopy offered a simpler way to develop robust HME processes to produce high quality products and allowing CQAs to be monitored in real time.

Theoretical and experimental approaches were used to address the possible reasons for the shift observed in the UV–Vis spectra from low to high (0.01 to 40 %) ITZ concentrations. The in-line UV–Vis spectra revealed a significant shift to higher wavelength from 0.01 to 0.1 %. DFT calculations at the B3LYP/6-31G* level revealed distinct behaviors among ITZ rotamers, with the coiled, higher-energy M0019 absorbing at lower wavelengths (324.36 nm) and the stable, planar M0373, with lower energy, absorbing at higher wavelengths (346.42 nm). This absorption pattern was further supported by IR spectra, where characteristic C—O stretching peaks (1072–977 cm^−1^) were more intense for the planar M0373 conformer. At low ITZ concentrations (1 %), FTIR spectral changes, such as broadening and peak shifts, indicated the presence of M0019, while higher concentrations (20 %) aligned with M0373, confirming concentration-dependent shifts between coiled and planar conformations.

No difference in the in-line and at-line UV–Vis spectra between the 1 and 20 % ITZ-KVA64-ASDs produced under static and shear conditions was found. Interestingly, the shift to a higher wavelength was detected even for the ITZ-KVA64-ASDs produced under static conditions. It can be suggested that shear from the extrusion was not the reason for the shift in UV as the concentration of ITZ increases. This work provides evidence to support that the shift observed in the in-line UV–Vis spectra from low (0.01 to 1 %) to high (20 to 40 %) ITZ concentration within the ITZ-KVA64-ASD system was mainly due to the conformational changes occurring in the ITZ structure. This work presents novel outcomes not yet reported for the ITZ-KVA64-ASD system.

## Funding

This work was financially supported by 10.13039/501100000601De Montfort University: the ‘Highflyer Scholarship programme 2018 to support Hetvi Triboandas PhD project.

## CRediT authorship contribution statement

**Hetvi Triboandas:** Writing – review & editing, Writing – original draft, Visualization, Validation, Methodology, Investigation, Formal analysis. **Mariana Bezerra:** Writing – review & editing, Writing – original draft, Software, Methodology. **Juan Almeida:** Writing – review & editing, Validation, Software, Methodology, Formal analysis, Data curation. **Matheus de Castro:** Writing – review & editing, Writing – original draft, Visualization, Methodology. **Bianca Aloise Maneira Corrêa Santos:** Writing – review & editing, Writing – original draft, Validation, Software, Methodology, Formal analysis. **Walkiria Schlindwein:** Writing – review & editing, Writing – original draft, Supervision, Resources, Project administration, Conceptualization.

## Declaration of competing interest

The authors declare that they have no known competing financial interests or personal relationships that could have appeared to influence the work reported in this paper.

## Data Availability

Data will be made available on request.

## Appendix A Supplementary data

Statistical analysis of the DoEs 1&2 and DSC and XRD results Supplementary material
