# Supplementary material for: Optimizing extrusion processes and understanding conformational changes in itraconazole amorphous solid dispersions using in-line UV–Vis spectroscopy and QbD principles
Source: Int J Pharm X. 2024 Nov 26;8:100308. doi: 10.1016/j.ijpx.2024.100308 (PMC11647160; doi:10.1016/j.ijpx.2024.100308)

***Supplementary material***

**Optimising Extrusion Processes and Understanding Conformational Changes in Itraconazole Amorphous Solid Dispersions Using In-Line UV-Vis Spectroscopy and QbD Principles**

**Hetvi Triboandas ^a^,**

**Mariana Bezerra ^b^,**

**Juan Almeida ^c^,**

**Matheus de Castro ^a^,**

**Bianca Aloise Maneira Corrêa Santos ^a^ and**

**Walkiria Schlindwein ^a, *^**

^a^ Leicester School of Pharmacy, De Montfort University, Leicester LE1 9BH, UK;

^b^ GlaxoSmithKline, David Jack Centre, Harris Lane, Ware, Hertfordshire SG12 0GX, UK;

^c^ Applied Materials, Daresbury, WA4 4AB, UK

Corresponding author: Walkiria Schlindwein, Leicester School of Pharmacy, Leicester LE1 9BH, UK, Tel.: +44 (116) 257 7124, England. E-mail: [wss@dmu.ac.uk](mailto:wss@dmu.ac.uk)

Abstract

In this supplementary section we report additional results for the statistical analysis of the DoEs 1&2 and DSC and XRD results for the respective DoEs. In addition, we also present the DSC and XRD for the verification samples. 15 local minima rotamers were generated using the MMFF force field and subsequently refined through PM6 semiempirical geometry optimization.

**Table S1**. Statistical analysis of DoE1 for the responses: a) 370 nm, b) 390 nm and c) L*. For each response the summary of fit, ANOVA and sorted parameter estimates are presented.

a)


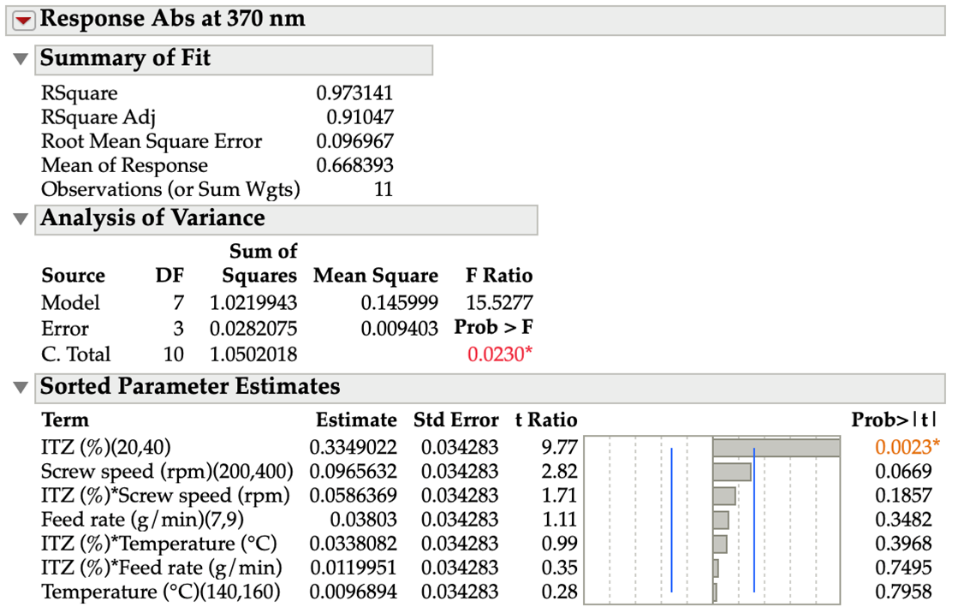


b)


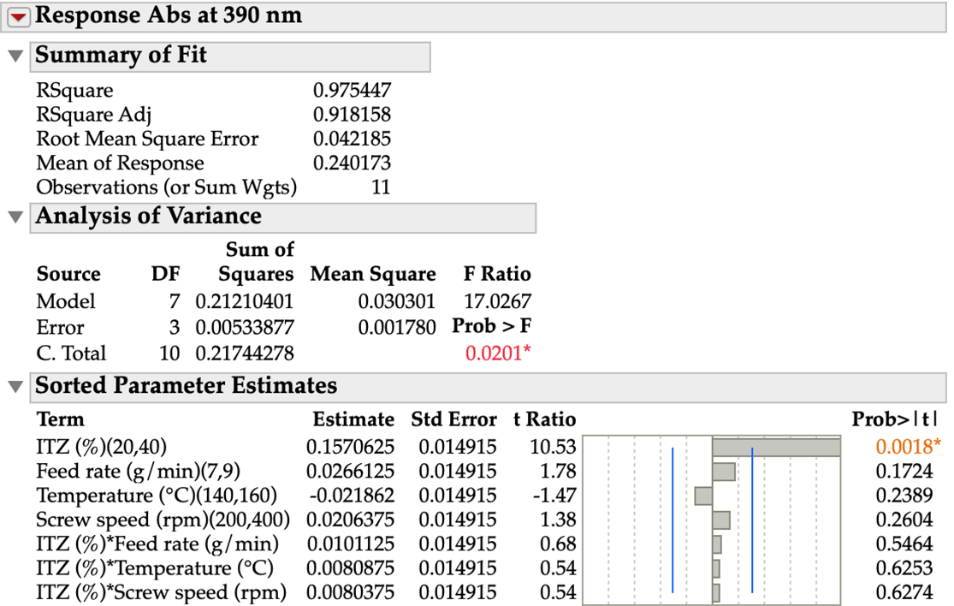


c)


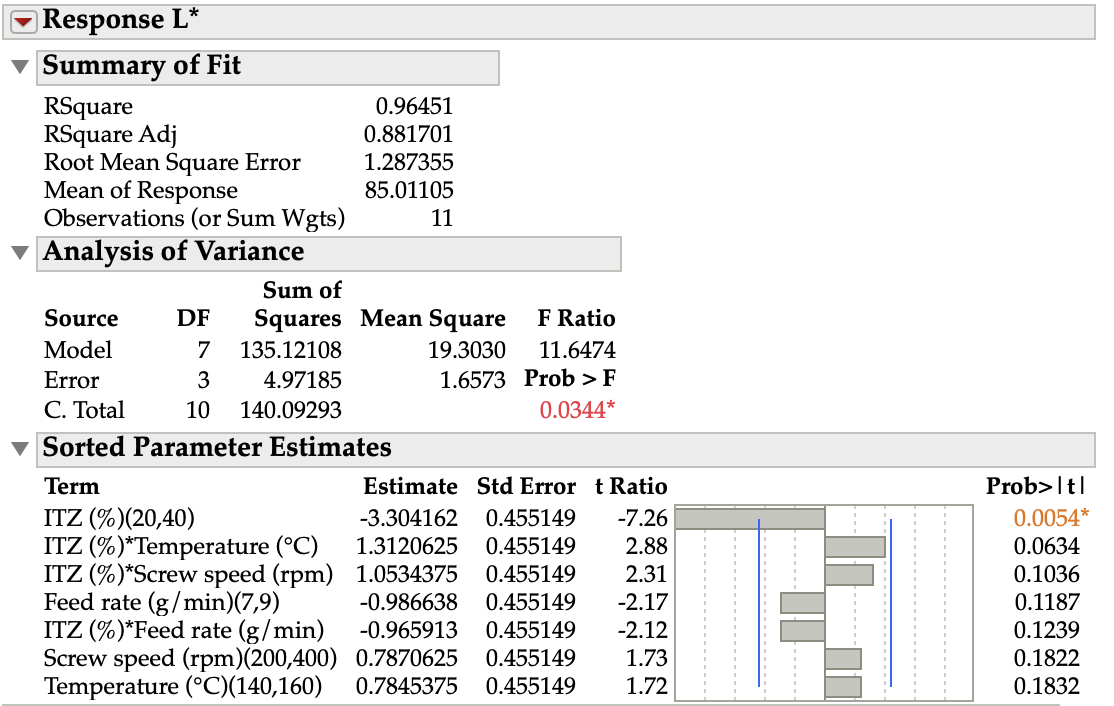


**Table S2**. Statistical analysis of DoE2 for the responses: a) 370 nm, b) 390 nm, c) L* and d) average torque. For each response the summary of fit, ANOVA and sorted parameter estimates are presented.

a)


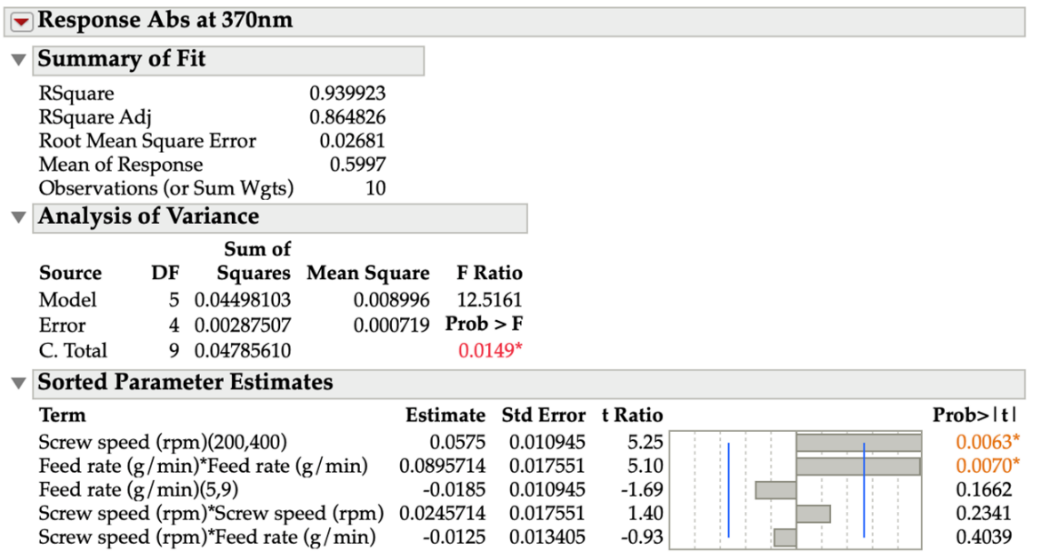


b)


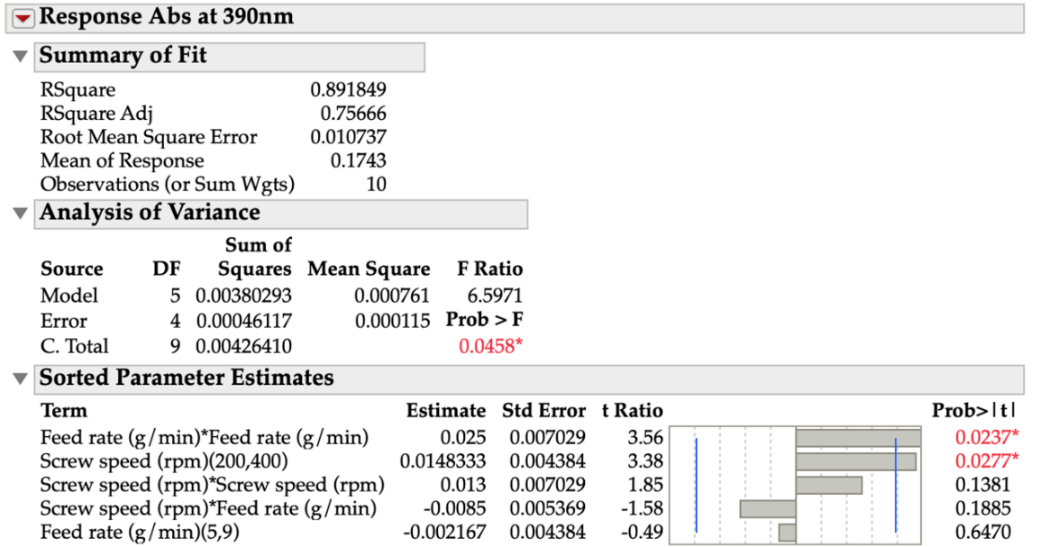


c)


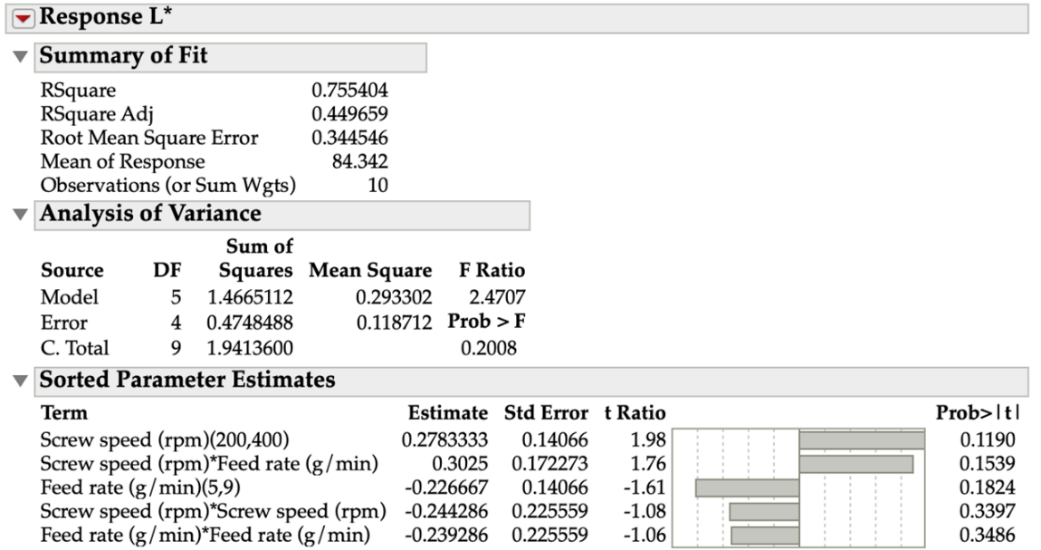


d)


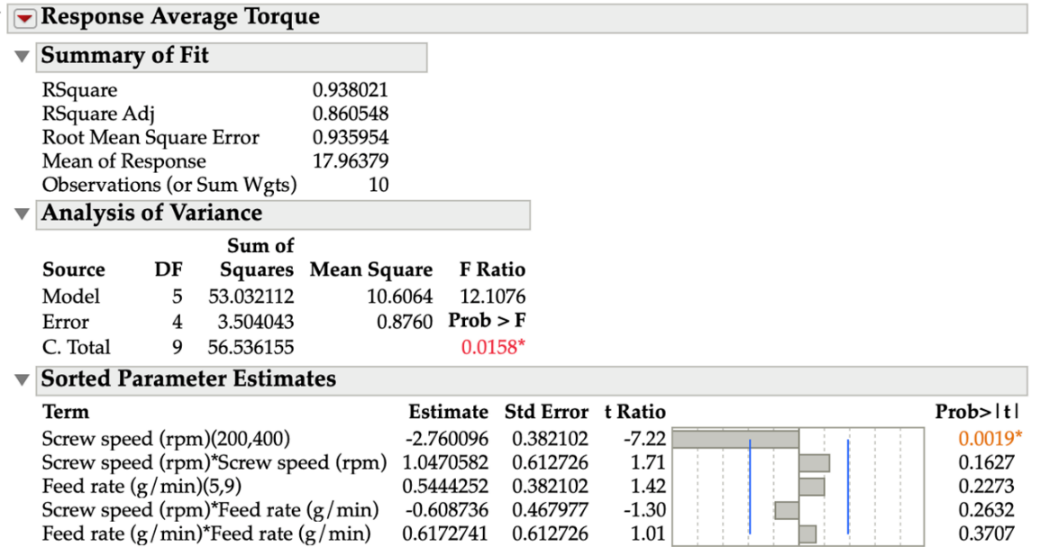


**Table S3** First excited states (S_0_ 🡪 S_1_ transition) computed using TD-DFT approach in vacuo (B3LYP/6-31G* level of theory) for the local minimum geometries selected of ITZ. The six lowest energy rotamers which are analyzed further in the study, are highlighted in bold.

| **Rotamer** | **DFT Energy (kJ/mol)** | **S_0_ 🡪 S_1_ transition (nm)** | **Dihedral º**  **(C36, C26, C25, C31)** |
| --- | --- | --- | --- |
| **M0373** | **-7918165.26** | **346.42** | **1.00** |
| **M0347** | **-7918158.64** | **370.25** | **0.14** |
| **M0337** | **-7918153.32** | **368.56** | **0.83** |
| **M0046** | **-7918150.41** | **322.41** | **-7.09** |
| **M0051** | **-7918150.28** | **321.35** | **-7.47** |
| **M0019** | **-7918148.67** | **324.56** | **6.85** |
| M0085 | -7918148.62 | 314.38 | 11.57 |
| M0268 | -7918146.78 | 338.78 | -0.39 |
| M0030 | -7918143.89 | 323.90 | 7.29 |
| M0071 | -7918143.10 | 329.35 | 10.18 |
| M0282 | -7918142.30 | 317.30 | -8.26 |
| M0319 | -7918141.50 | 325.48 | -12.43 |
| M0280 | -7918138.23 | 321.76 | -6.04 |
| M0382 | -7918136.91 | 327.44 | -9.05 |
| M0158 | -7918135.56 | 336.23 | -7.67 |

**Fig. S1** a) Differential scanning calorimetry for DoEs 1&2; b) Powder X-Ray Diffraction for DoEs 1&2. The notation in the legend is: run number_ ITZ concentration_temperature_screw speed_feed rate.

1. DoE1
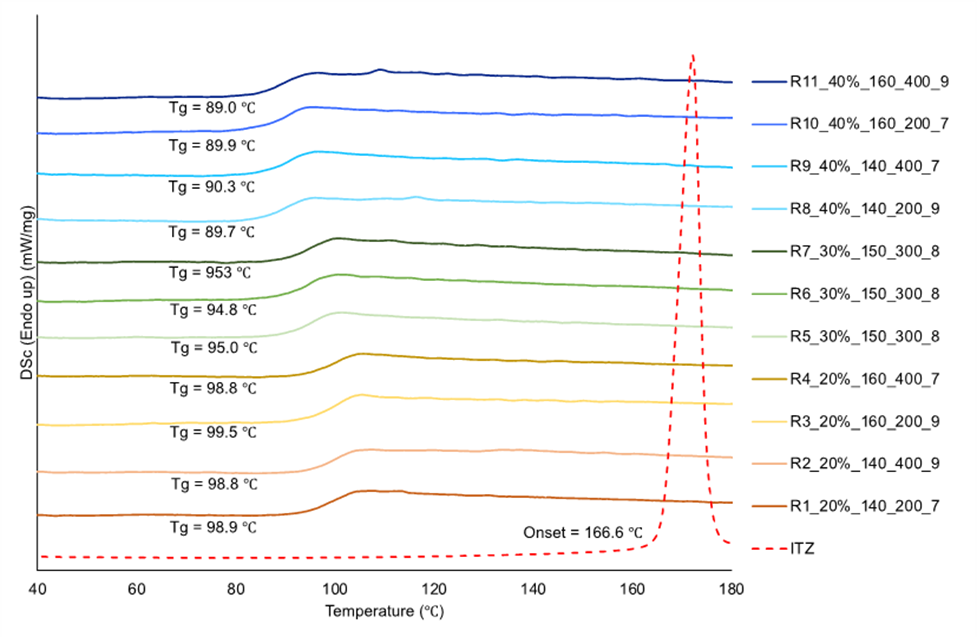


DoE2


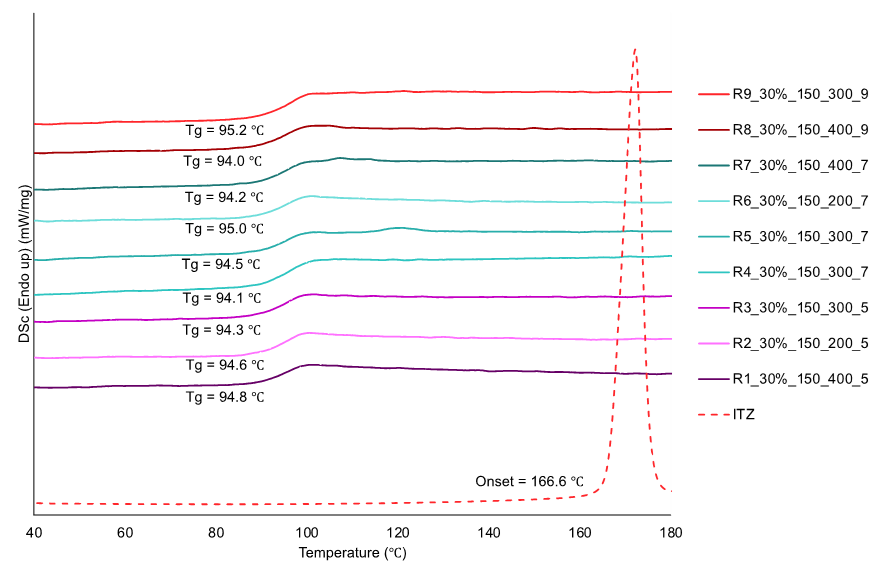


1. DoE 1


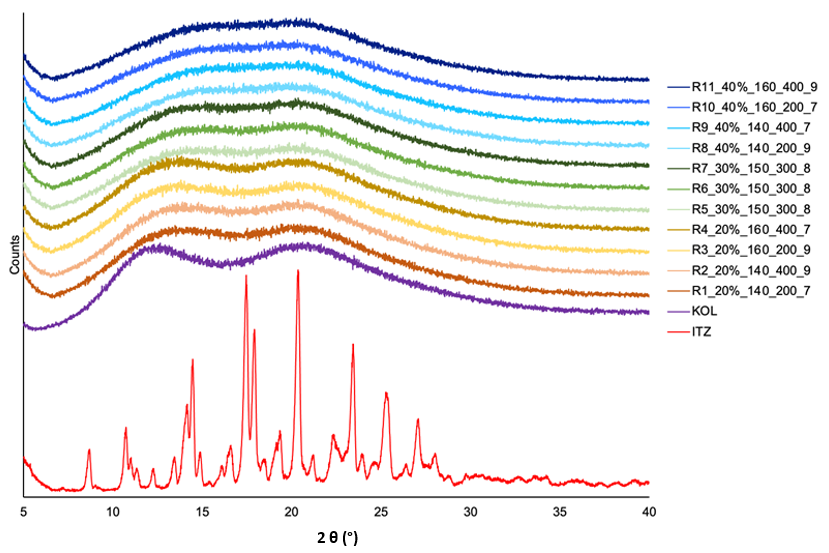


DoE2


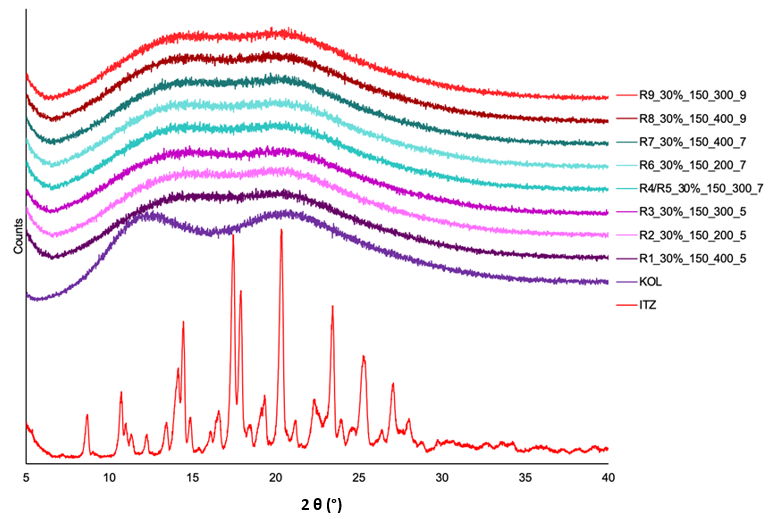


**Fig. S2**. a) Differential scanning calorimetry and b) Powder X-Ray Diffraction for the four verification runs R1-R4 inside the operation design space.

a)


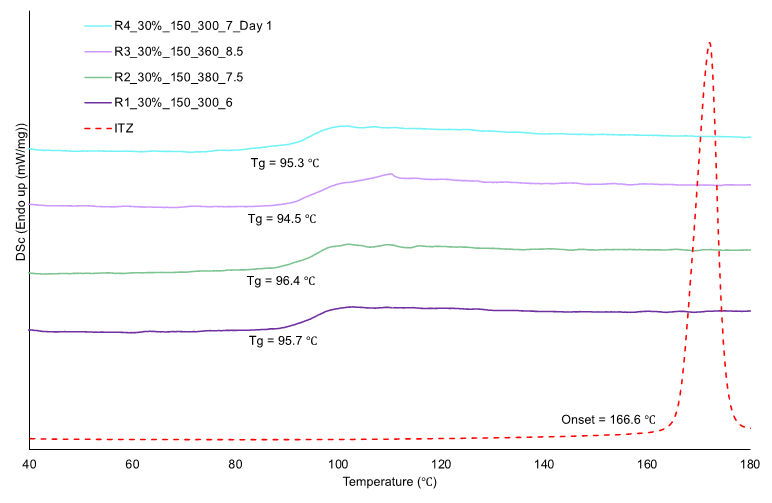


b)


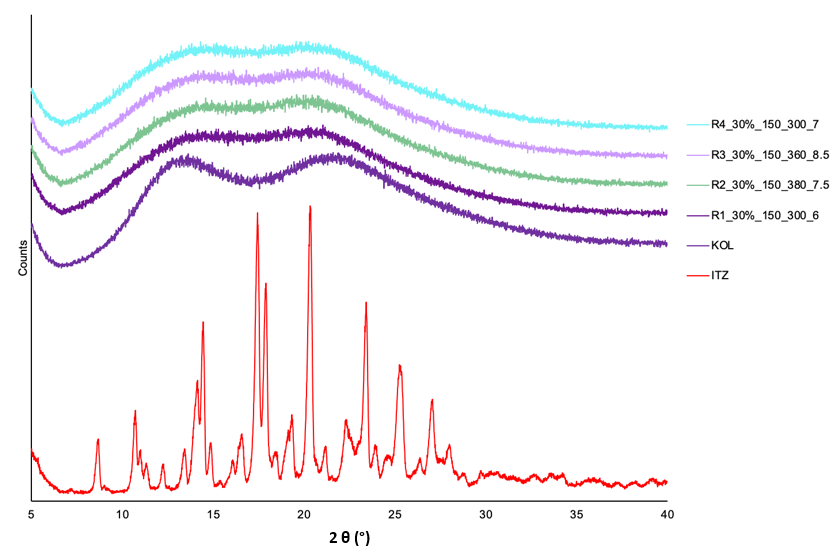

Supplement: Statistical analysis of the DoEs 1&2 and DSC and XRD results [file mmc1.docx]
